# Biomarker Modulation by Aspirin and Metformin for Colorectal Cancer Prevention: Results from the ASAMET Randomized Phase II Trial

**DOI:** 10.1158/2767-9764.CRC-26-0556

**Published:** 2026-11-04

**Authors:** Irene M. Briata, Alessio Carbone, Sebastian Foersch, Katrin E. Tagscherer, Wilfried Roth, Dominique Scherer, Tania Buttiron Webber, Matteo Clavarezza, Jürgen Burhenne, Martino Oliva, Mauro D’Amico, Giorgia Giancarli, Massimo Oppezzi, Paola Romagnoli, Mauro Dalla Libera, Borut Stabuc, Thomas Bachleitner-Hofmann, Marilena Petrera, Laura Paleari, Matteo Puntoni, Cornelia M. Ulrich, Andrea DeCensi

**Affiliations:** 1 Division of Medical Oncology, E.O. Galliera Hospital, Genoa, Italy.; 2 Institute of Pathology, University Medical Centre, Mainz, Germany.; 3 Statistical Genetics Research Group, Institute of Medical Biometry, Heidelberg University, Heidelberg, Germany.; 4 Department of Clinical Pharmacology and Pharmacoepidemiology, Heidelberg University Hospital, Heidelberg, Germany.; 5 Division of Gastroenterology, E.O. Galliera Hospital, Genoa, Italy.; 6 Department of Gastroenterology, University Medical Centre Ljubljana, Ljubljana, Slovenia.; 7 Division of Visceral Surgery, Department of General Surgery, Medical University of Vienna, Vienna, Austria.; 8 Clinical and Epidemiological Research Unit, University Hospital of Parma, Parma, Italy.; 9 Huntsman Cancer Institute and Department of Population Health Sciences, University of Utah, Salt Lake City, Utah.; 10 Clinical Centre, Champalimaud Foundation, Lisbon, Portugal.

## Abstract

**Purpose:**

Aspirin (Asa) and metformin (Met) have shown preventive efficacy in colorectal cancer. Preclinical data suggest convergence on NF-κB/PI3K–mTOR signaling and potential synergy. The ASAMET phase II trial tested this hypothesis in patients with resected stage I to III colorectal cancer.

**Patients and Methods:**

In a 2 × 2 factorial, multicenter, double-blind study (EudraCT 2015-004824-77; NCT03047837), 51 patients were randomized to placebo, Asa 100 mg once daily, Met 850 mg twice daily, or AsaMet for 12 months. Biopsies of normal colonic mucosa and fasting blood samples were obtained at baseline and 12 months. NF-κB (primary endpoint), Ki67, EGFR, and pS6K were assessed by IHC.

**Results:**

After 12 months, compared with placebo, Met reduced global NF-κB by −9.04 [95% confidence interval (CI), −17.55 to −0.54; *P* = 0.04], whereas Asa alone was inactive (−3.58; 95% CI, −12.15 to 4.99; *P* = 0.41), and AsaMet showed a nonsignificant reduction (−8.88; 95% CI, −18.86 to 1.10; *P* = 0.08). Pooled analyses confirmed a Met-driven effect (Met vs. non-Met: −7.29; 95% CI, −13.79 to −0.79; *P* = 0.03), with no impact of Asa (*P* = 0.73). Ki67 and pS6K were unchanged globally; however, AsaMet increased right-sided Ki67 (12.48; 95% CI, 0.45–24.51; *P* = 0.04). No treatment modulated pS6K. Toxicity was manageable, and no serious adverse events occurred.

**Conclusions:**

Met reduces NF-κB expression in normal colorectal mucosa of survivors of colorectal cancer, supporting its potential as a low-toxicity preventive agent. Asa did not favorably affect mucosal biomarkers. These findings support tissue-based endpoints and further evaluation of Met for colorectal cancer prevention.

**Significance:**

The ASAMET trial provides the first randomized evidence that Met significantly modulates NF-κB expression in histologically normal colorectal mucosa of survivors of colorectal cancer. This finding supports a biologically plausible mechanism of action in secondary cancer prevention that could be exploited in the postadjuvant setting. Asa, by contrast, did not favorably affect any mucosal biomarker endpoint in this population. These results may support a phase III study of Met in this setting.

**Trial Registration:**

ClinicalTrials.gov Identifier: NCT03047837; EudraCT number: 2015-004824-77.

## Introduction

Colorectal cancer is the third most commonly diagnosed malignancy and the second leading cause of cancer-related death worldwide, with more than 1.9 million new cases and approximately 930,000 deaths estimated in 2020 alone (1). Projections from GLOBOCAN 2020 estimate that colorectal cancer will exceed 3.2 million new cases and 1.6 million deaths globally by 2040, a 63% increase mainly driven by aging and population growth in high-income regions (2). Notably, an increasing burden is also observed among younger adults under 50 years of age, with early-onset colorectal cancer showing a steady increase in incidence, particularly in Western countries (2).

Although improvements in surgical techniques, adjuvant treatments, surveillance, and prevention strategies (3) have improved survival rates, recurrence remains a major concern with a 5-year recurrence risk of approximately 30% to 40% in patients with stage I to III colorectal cancer (4). This highlights an urgent need for effective and safe interventions in the adjuvant and prevention settings, especially capable of targeting molecular pathways involved in disease progression.

Among pharmacologic agents with potential preventive properties, aspirin (Asa) and metformin (Met) have gained considerable attention. Asa, widely used in cardiovascular disease prevention, has demonstrated significant anticancer effects in both epidemiologic and interventional studies. In particular, low-dose Asa has been shown to reduce colorectal cancer incidence, recurrence, and mortality, with stronger effects observed in patients with *PIK3CA* mutations or tumors originating in the proximal colon (5–7), yet efficacy seems to be context-dependent. In the international ASCOLT phase III trial (*n* = 1,550), 200 mg Asa daily for 3 years after standard adjuvant therapy failed to improve disease-free survival (HR, 0.91; *P* = 0.38; ref. 8). Conversely, the Scandinavian ALASCCA study showed that 160 mg Asa daily for 3 years halved the 3-year recurrence risk in patients with PI3K pathway–altered colorectal cancer (HR, 0.49–0.42 across genomic subgroups; ref. 9). Met, the most commonly prescribed treatment for type 2 diabetes, has demonstrated antiproliferative and proapoptotic effects in preclinical colorectal cancer models, and several observational studies have associated its use with lower recurrence in diabetic patients with colorectal cancer (10, 11).

Notably, a phase III randomized trial in nondiabetic patients with a history of colorectal adenomas showed that 1 year of low-dose Met (250 mg/day) significantly reduced the prevalence and number of metachronous adenomas and polyps compared with placebo, suggesting a potential chemopreventive effect of Met (12). Mechanistically, Asa exerts anti-inflammatory effects through irreversible inhibition of cyclooxygenase enzymes and modulation of NF-κB signaling, a transcription factor central to inflammation and tumorigenesis (13). A recent study has shown that Asa’s antimetastatic activity also stems from lifting platelet-derived thromboxane A_2_ (TXA_2_)–mediated suppression of cytotoxic T cells, thereby enhancing immune elimination of disseminating cancer cells (14). Met activates AMP-activated protein kinase (AMPK), leading to inhibition of the mTOR pathway, reduced insulin/insulin-like growth factor-1 signaling, and decreased cellular proliferation (15). Preclinical data suggest a potential synergistic interaction between Asa and Met, converging on key oncogenic pathways such as PI3K/AKT/mTOR and NF-κB (16, 17). *In vitro* and *in vivo* studies have shown that the combination can enhance apoptotic responses, reduce tumor growth, and inhibit pro-inflammatory mediators more effectively than either agent alone (18). Recent work in *PIK3CA*-mutant colorectal cancer cells confirmed additive cytotoxic and colony-inhibitory effects at clinically achievable concentrations (19). Collectively, these data provide a strong biological rationale for evaluating Asa and Met—alone and in combination—as low-toxicity, biomarker-guided interventions to test this synergistic hypothesis in a clinical setting.

Therefore, the ASAMET trial was designed to evaluate the effect of a 12-month treatment with Asa (100 mg/day) and Met (850 mg twice daily), alone or in combination, in patients with resected stage I to III colorectal cancer. In this report, we present the effects of treatment on key tissue biomarkers—NF-κB, the primary endpoint, and Ki67 and pS6K, assessed in histologically normal (unaffected) colonic mucosa of both sides, as surrogate indicators of field cancerization inhibition.

## Patients and Methods

### Study design and oversight

The ASAMET trial (NCT03047837 and EudraCT 2015-004824-77) was a multicenter, randomized, double-blind, placebo-controlled phase II study that tested Asa and Met in a 2 × 2 factorial design. The study used a double-dummy design to maintain blinding. Randomization was performed using a web-based centralized system with a permuted block design. Stratification factors included study center and (neo-)adjuvant chemotherapy (yes vs. no). Allocation was concealed through the centralized randomization system. Participants, investigators, and outcome assessors were blinded to treatment allocation. The study design and rationale have been published previously (20). Eligible patients with resected colorectal cancer (stage I–III) within 24 months of surgery were randomized to one of four arms in a 2 × 2 factorial design:

- Arm A: placebo Met + placebo Asa.
- Arm B: Met 850 mg twice daily.
- Arm C: Asa 100 mg/day.
- Arm D: Asa 100 mg/day + Met 850 mg twice daily.

Key inclusion criteria included age 18 to 80 years, completely resected stage I to III colorectal cancer, and Eastern Cooperative Oncology Group performance status ≤1. Treatment was administered for 12 months. Patients were seen every 3 months for blood biomarkers, treatment compliance, and toxicity according to Common Terminology Criteria for Adverse Events version 4. Colonoscopy biopsies and blood samples were collected at baseline and at 12 months. The primary endpoint was the absolute change in NF-κB expression by IHC in normal colonic tissue of both colon sides. In addition to the primary endpoint, changes in the IHC expression of pS6K, Ki67, and EGFR in normal tissue were evaluated as secondary tissue endpoints. Additional circulating biomarkers and Met serum concentrations will be reported separately.

The study was approved by the relevant ethics committees in Italy, Slovenia, and Austria (Division of Medical Oncology, E.O. Galliera Hospital, Genoa; Department of Gastroenterology, University Medical Centre Ljubljana; and Department of General Surgery, Medical University of Vienna) and was run in accordance with the Declaration of Helsinki and International Council for Harmonisation Good Clinical Practice guidelines. All participants gave written informed consent.

### Biopsy collection and IHC analysis

At baseline and after 12 months of treatment, participants underwent colonoscopy with targeted biopsies of macroscopically normal mucosa both in the right colon and the left colon. In patients who received right colectomy, 6 biopsies were taken at the level of the cul de sac and 6 biopsies 2 cm above the third Houston valve. In patients operated for a left side colonic cancer, 6 biopsies were collected near the ileocecal valve and 6 biopsies 2 cm above the third Houston valve or 2 cm above the anastomosis in patients operated for rectal cancer. Fasting blood samples were also collected at baseline and 12 months after colonoscopy.

Formalin-fixed, paraffin-embedded colonic biopsy sections were centrally stained for the p65 (RelA) subunit of NF-κB, pS6K, and Ki67 using a validated automated IHC protocol. Digital image analysis was carried out at the Institute of Pathology, University Medical Center Mainz, as part of the ASAMET translational study. Primary antibodies against NF-κB (#8242S, RRID: AB_2797994) and pS6K (#35708, RRID: AB_2936859) were obtained from Cell Signaling Technology and used at a final concentration of 1:10,000 and 1:250, respectively. The Ki67 antibody (#IR62661-2, RRID: AB_2631211) was purchased from Agilent Technologies as ready to use. IHC staining was carried out on an automated staining platform (Lab Vision Autostainer 480s, Thermo Fisher Scientific). Stained slides were digitalized using the NanoZoomer 2.0-HT System (Hamamatsu) and analyzed using the open-source digital image software QuPath (PMID: 29203879, RRID: SCR_018257). For NF-κB and pS6K, both cytoplasmic and nuclear staining was evaluated. For Ki67, only nuclear staining was assessed. Results are expressed as the percentage of positively stained cells relative to total number of cells counted.

### Statistical analysis

The original target sample size of 160 patients (40 per arm) was calculated to provide 80% power to detect a synergistic effect of Asa + Met and 90% power for each monotherapy, assuming a two-sided α of 0.05, as described previously (20). The sample size calculation accounted for an expected 10% dropout rate. However, because of recruitment challenges during COVID-19 pandemic and logistic constraints, the trial enrolled fewer patients. Consequently, all analyses presented here should be interpreted as hypothesis-generating and consistent with the objectives of an exploratory study.

All analyses were conducted on the per-protocol population, defined as all randomized patients with both baseline and 12-month paired biomarker data. Differences in tissue biomarker expression across the four treatment groups (placebo Asa + placebo Met, Asa, Met, and Asa + Met) were evaluated using two-way analysis of covariance (ANCOVA), with fixed factors for Met (yes/no), Asa (yes/no), and their interaction. Baseline biomarker levels and biopsy site (right vs. left colon) were included as covariates. Assumptions for ANCOVA were assessed using the Shapiro–Wilk test for normality and the Levene test for homogeneity of variances. Residual diagnostics were also examined. When model assumptions were violated, robust regression models were applied with heteroscedasticity-consistent standard errors. Outliers were handled conservatively to minimize their influence on parameter estimates.

To increase power and interpretability and given the lack of interaction between drugs, two prespecified subgroup comparisons were also conducted:

- Asa versus no Asa: The AsaMet and Asa arms were combined and compared with the no Asa group, which included the placebo and Met arms;
- Met versus no Met: The Met and AsaMet arms were combined and compared with the no Met group, consisting of the placebo and Asa arms.

**Figure 1.**
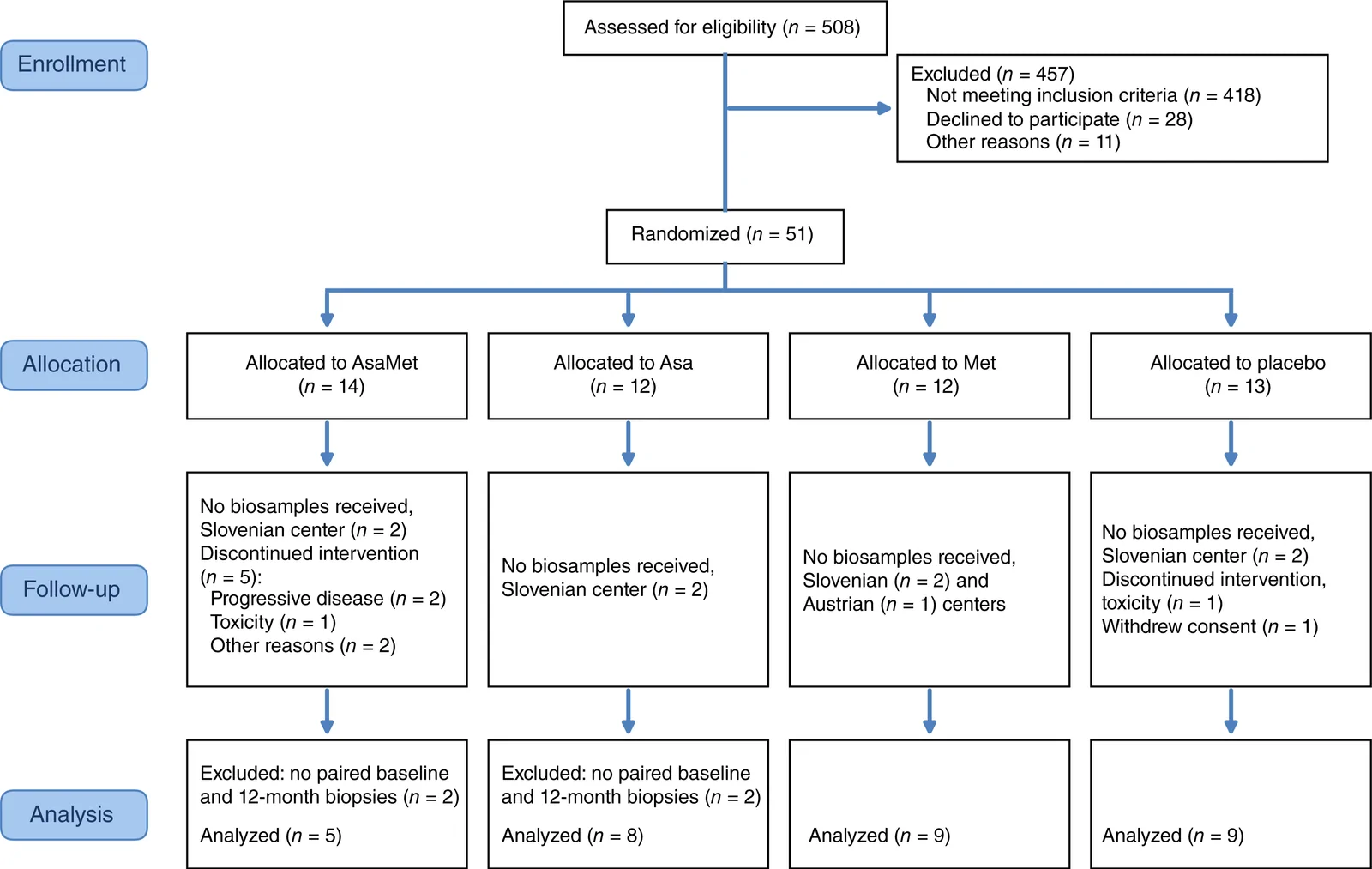
Participant flow diagram including surrogate endpoints.

These binary groupings allowed focused evaluation of each treatment independently. All analyses were adjusted for baseline biomarker values and tumor location. The dataset was extensively cleaned and verified prior to analysis. Sex was considered as a potential biological variable; the study population comprised 32 males (62.7%) and 19 females (37.3%), and all primary analyses were conducted in the overall cohort without stratification by sex. Participant demographics at baseline are summarized in Table 1; mean age across arms ranged from 63.4 to 68.3 years and mean body mass index from 24.1 to 26.8 kg/m^2^. Attrition and protocol deviations are depicted in the CONSORT flow diagram (Fig. 1); of the 51 randomized patients, 31 had paired baseline and 12-month biomarker data and were included in the per-protocol analysis. All statistical procedures were performed using Stata/SE version 19 (StataCorp; RRID: SCR_012763).

**Table 1.** Main baseline patient characteristics.

Characteristic	Asa (*N* = 12)	Met (*N* = 12)	AsaMet (*N* = 14)	Pla (*N* = 13)	*P* value
Age at randomization, years	67.7 (5.1)	67.7 (9.2)	63.4 (10.2)	68.3 (6.7)	0.38
Gender	​	​	​	​	0.10
Male	11 (91.7)	7 (58.3)	7 (50)	7 (53.9)	
Female	1 (8.3)	5 (41.7)	7 (50)	6 (46.2)	
Performance status	​	​	​	​	0.72
0	11 (91.7)	12 (100)	14 (100)	12 (100)	
1	1 (8.3)	—	—	—	
Body mass index^a^, kg/m^2^ by threshold	​	​	​	​	0.98
<25	5 (41.7)	5 (41.7)	8 (57.1)	5 (38.5)	
25–30	5 (41.7)	5 (41.7)	4 (28.6)	6 (46.2)	
>30	2 (16.7)	2 (16.7)	2 (14.3)	2 (15.4)	
Surgery type	​	​	​	​	0.29
Left colectomy	2 (16.7)	6 (50)	6 (42.9)	5 (41.7)	
Right colectomy	2 (16.7)	3 (25)	4 (28.6)	2 (16.7)	
Total Mesorectal Excision (TME)	2 (16.7)	—	3 (21.4)	3 (25)	
Other	6 (50)	3 (25)	1 (7.1)	2 (16.7)	
Stage	​	​	​	​	0.90
I or II	7 (63.6)	6 (50)	8 (57.1)	6 (50)	
III	4 (36.4)	6 (50)	6 (42.9)	6 (50)	
Grading	​	​	​	​	0.61
G1 or G2	7 (63.6)	8 (66.7)	5 (41.7)	7 (58.3)	
G3 or G4	4 (36.4)	4 (33.3)	6 (50)	3 (25)	
GX	—	—	1 (8.3)	2 (16.7)	
Previous chemotherapy	​	​	​	​	0.86
Yes	7 (58.3)	9 (75)	9 (64.3)	7 (58.3)	
No	5 (41.7)	3 (25)	5 (35.7)	5 (41.7)	
Previous radiotherapy	​	​	​	​	1
Yes	2 (18.2)	2 (16.7)	3 (21.4)	3 (25)	
No	9 (81.8)	10 (83.3)	11 (78.6)	9 (75)	

Mean (SD) or *n* (%).

a Body mass index: <25 normal weight; 25–30 over weight; and >30 obese.

## Results

A total of 51 patients were randomized in the ASAMET trial: 42 in Italy, 8 in Slovenia, and 1 patient in Austria. The CONSORT flow diagram is depicted in Fig. 1. Baseline characteristics of the study population are summarized in Table 1. No statistically significant differences between the four treatment arms were detected, confirming the comparability of the groups at baseline.

The baseline expression levels of key biomarkers (NF-κB, Ki67, pS6K, and EGFR) in normal colonic tissue were analyzed and are presented in Supplementary Table S1. These biomarkers were also assessed separately for the right and left colonic tissue. The median values ranged, respectively, from 44.7% to 57% for NF-κB, 15.3% to 27.7% for Ki67, and >70% for pS6K. No statistically significant differences were observed at baseline across the treatment groups for any of the biomarkers.

**Figure 2.**
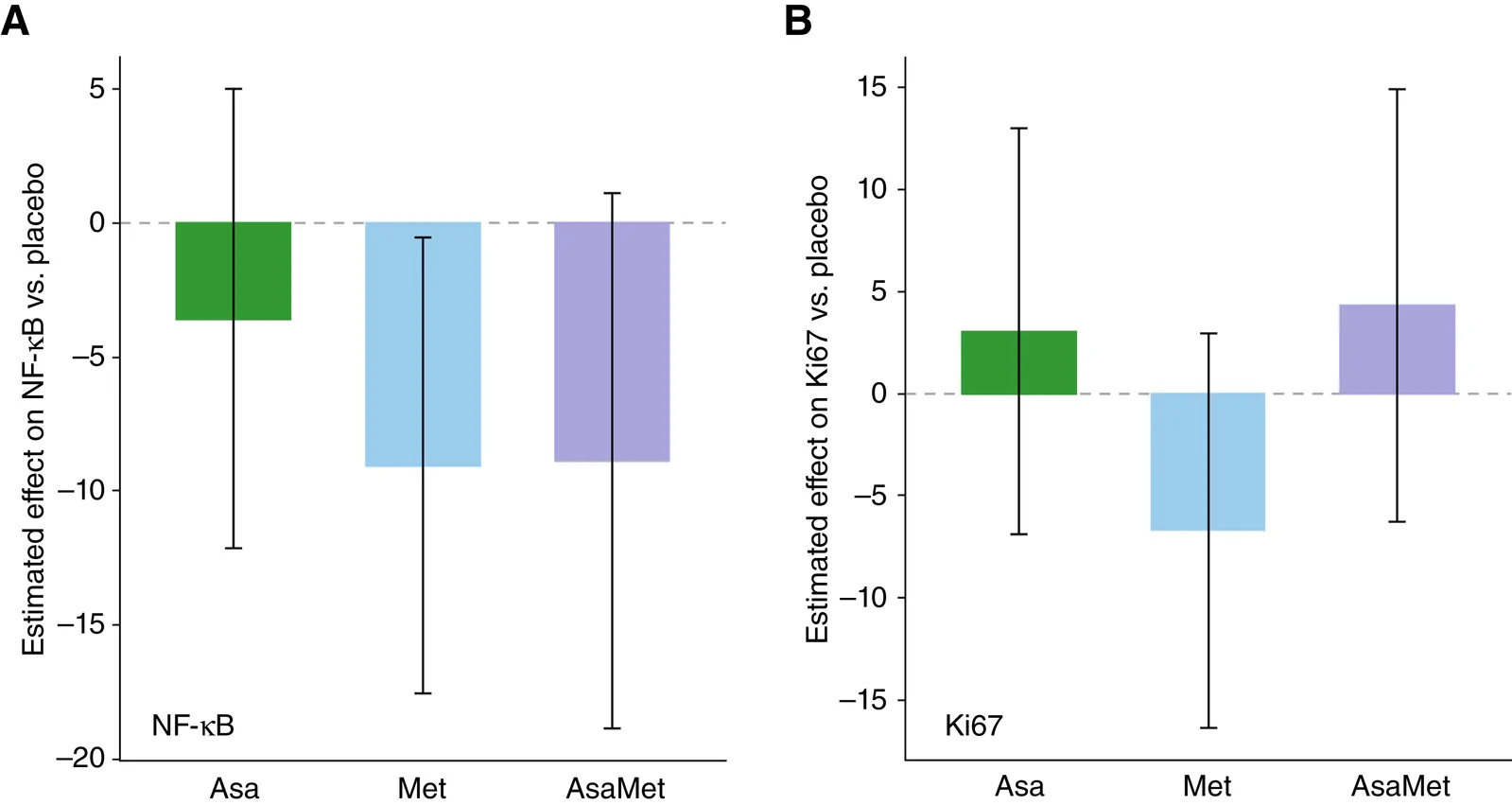
Overall tissue changes of NF-κB (**A**) and Ki67 (**B**) according to the treatment arm compared with placebo.

After 12 months, Met produced a significant reduction in NF-κB versus placebo in overall colonic tissue [−9.04; 95% confidence interval (CI), −17.55 to −0.54; *P* = 0.04] and Asa alone was inactive (−3.58; 95% CI, −12.15 to 4.99; *P* = 0.41), whereas the combination (AsaMet) showed a nonsignificant trend of reduced expression (−8.88; 95% CI, −18.86 to 1.10; *P* = 0.08; Fig. 2A). Global Ki67 did not differ significantly among treatments relative to placebo (Met: −6.70; 95% CI, −16.36 to 2.96; Asa: 3.04; 95% CI, −6.92 to 12.99; and AsaMet: 4.31; 95% CI, −6.27 to 14.90; all *P* ≥ 0.17; Fig. 2B).

**Figure 3.**
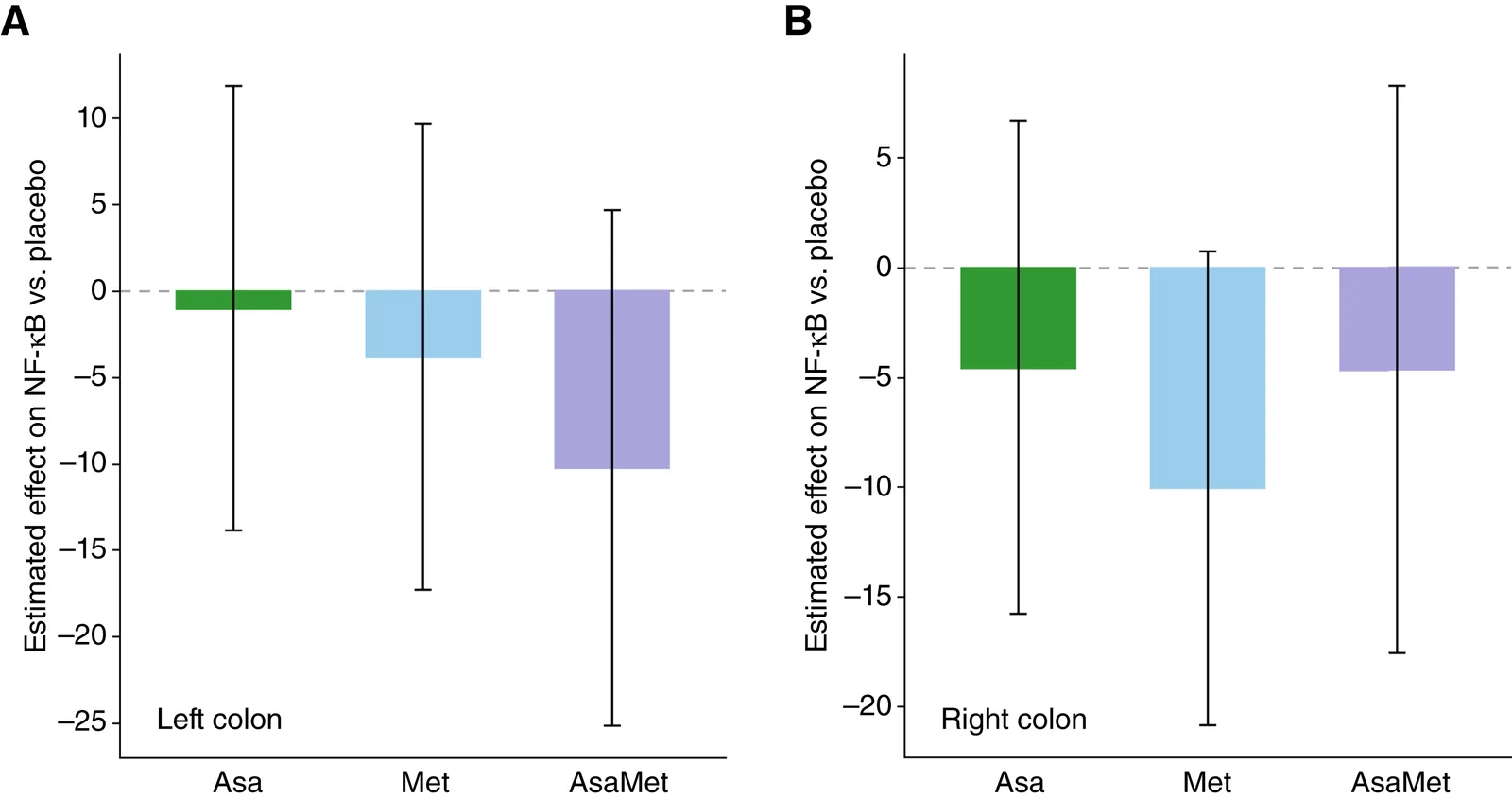
Tissue NF-κB changes in the left (**A**) and right (**B**) colon according to the treatment arm compared with placebo.

**Figure 4.**
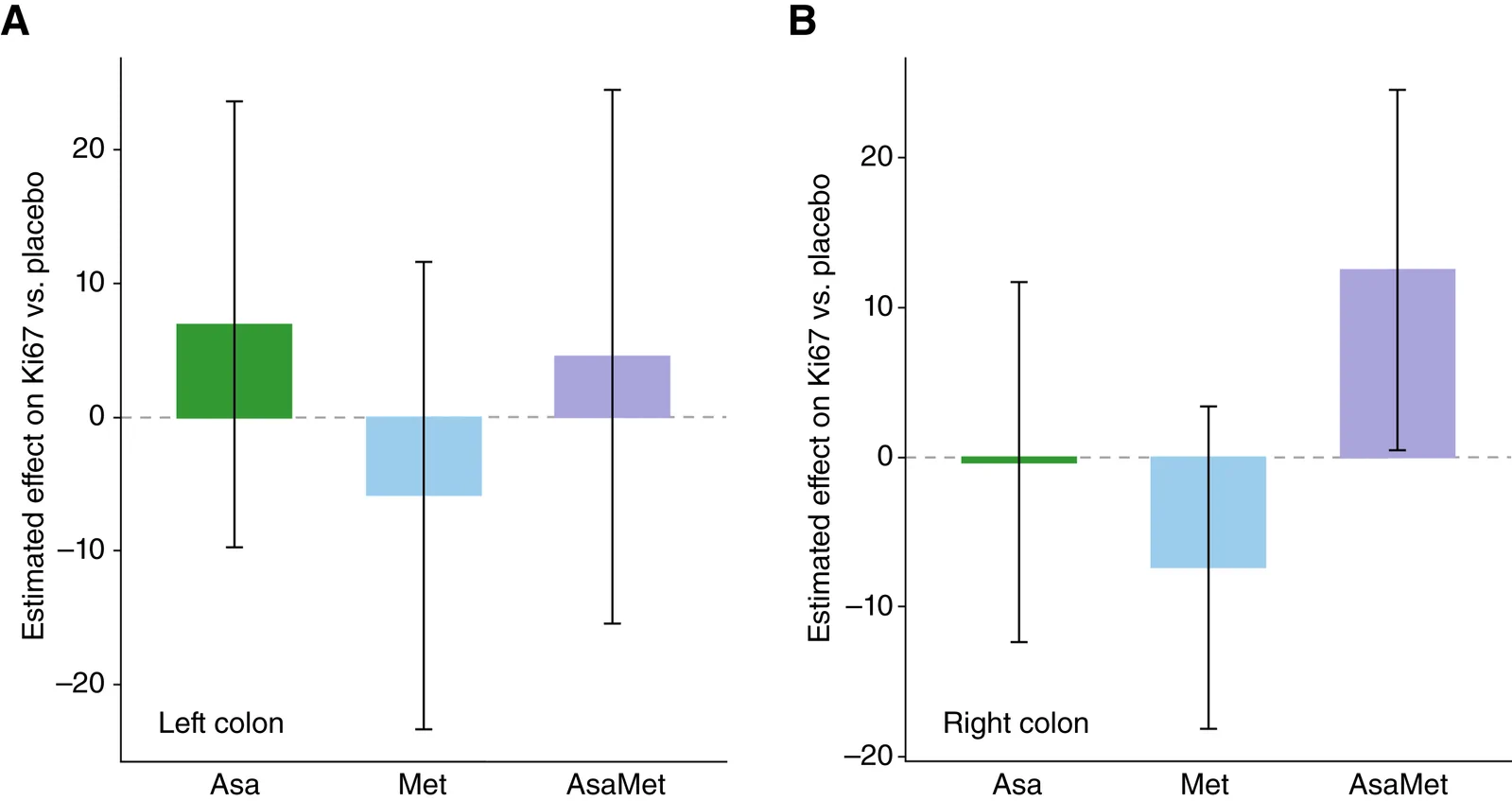
Tissue Ki67 changes in the left (**A**) and right (**B**) colon according to the treatment arm compared with placebo. Estimates for the right colon were obtained by robust regression.

Looking at colon site, Met lowered NF-κB in the right colon (−10.05; 95% CI, −20.85 to 0.75; *P* = 0.07) and to a lesser extent in the left colon (−3.82; 95% CI, −17.30 to 9.66; *P* = 0.57; Fig. 3A and B). Pooling the arms confirmed a Met-driven effect: Met-containing regimens (Met and AsaMet) reduced global NF-κB by 7.29 points versus non-Met (−7.29; 95% CI, −13.79 to −0.79; *P* = 0.03), whereas Asa versus no Asa showed no difference (*P* = 0.732; Supplementary Table S2). At colon-site level, Ki67 changes in the left colon were nonsignificant across arms (all *P* ≥ 0.398; Fig. 4A), whereas in the right colon, AsaMet increased Ki67 by 12.48 points compared with placebo (95% CI, 0.45–24.51; *P* = 0.043), with no effect of Met or Asa alone (*P* = 0.170 and *P* = 0.954, respectively; Fig. 4B). Pooling arms confirmed an Asa-driven effect: Asa-containing regimens increased Ki67 by 7.08 points versus non-Asa (7.08; 95% CI, −0.10 to 14.25; *P* = 0.05), whereas Met versus no Met showed no difference (*P* = 0.34; Supplementary Table S3). Neither global nor segmental pS6K expression was modified by any treatment. Point estimates ranged from −3.90 to 0.86 with *P* values between 0.36 and 0.96 for all contrasts.

Toxicity was manageable and in line with the known profile of both drugs (Table 2).

**Table 2.** Number of adverse events and grade by treatment arm.

​	Asa (*N* = 12)		Met (*N* = 12)		AsaMet (*N* = 14)		Pla (*N* = 13)	
	G1/2	G3	G1/2	G3	G1/2	G3	G1/2	G3
Events, *N*	​	​	​	​	​	​	​	​
Anorexia	—	—	—	—	3	—	—	—
Diarrhea	3	—	9	2	5	1	5	1
Dyspepsia	1	—	3	—	1	—	3	—
Flu-like symptoms	2	—	1	—	2	—	—	—
Gastrointestinal^a^	2	—	—	—	1	—	5	1
Nausea	—	—	6	—	1	—	1	—
Amylase increase	—	—	—	1	—	—	—	—
Other	8	—	3	—	12	—	5	—

a Nausea and abdominal pain.

## Discussion

This randomized, phase II trial provides new insights into the biological effects of low-dose Met and Asa, alone or in combination, in patients previously treated for nonmetastatic colorectal cancer. The primary finding was a statistically significant reduction in NF-κB expression in normal colonic mucosa after 12 months of Met treatment compared with placebo. This anti-inflammatory effect was observed globally in the colonic mucosa and remained significant when the two Met-containing arms were analyzed together. The effect of Met was also borderline significant in each colon site despite limited power. On the other hand, Asa, alone or in combination, did not produce significant changes in NF-κB, and its combination with Met was associated with an unexpected increase in Ki67 expression in right-sided mucosa.

NF-κB is a transcription factor implicated in inflammation-driven carcinogenesis, and its overexpression has been associated with colorectal tumor progression and recurrence (21, 22). The observed reduction in NF-κB with Met suggests a plausible anti-inflammatory mechanism of action, independent of glycemic control. This is consistent with preclinical evidence showing that Met suppresses NF-κB activation through AMPK-mediated inhibition of IKKβ and reduction of mitochondrial reactive oxygen species. Moreover, pharmacokinetic data from our previous pilot study in nondiabetic individuals have shown that Met accumulates in colonic tissue at concentrations approximately 150-fold higher than that in plasma, supporting a direct local effect on the colorectal mucosa (23). Our findings also align with those of Higurashi and colleagues (12), who demonstrated a reduced recurrence of colorectal adenomas in nondiabetic patients receiving low-dose Met for 1 year. Together, these results support the hypothesis that Met may exert chemopreventive activity by modulating mucosal inflammation in the normal colorectal epithelium.

In contrast, Asa showed no measurable benefit on NF-κB, Ki67, or pS6K expression globally. In fact, we noted an increase in Ki67. This absence of short-term biomarker modulation contrasts with long-term evidence from large observational and interventional studies. Rothwell and colleagues (5) reported that daily Asa use reduces colorectal cancer incidence and mortality, but only after 5 to 10 years of continued exposure. Similarly, the CAPP2 trial demonstrated a significant reduction in colorectal cancer incidence among carriers of Lynch syndrome treated with 600 mg/day Asa, with effects becoming apparent only after extended follow-up (24). The ASPREE trial, however, challenged this paradigm by showing an increased risk of cancer-related mortality in significantly older adults (≥70 years) receiving low-dose Asa with a much shorter follow-up (∼4.7 years) compared with prior studies among healthy individuals, including a nonsignificant trend toward increased colorectal cancer incidence (25). Thus, Asa was initiated late in life and administered for a relatively short period. The trial duration and follow-up were well suited to detect short-term benefits and harms (e.g., bleeding and mortality) but not long-latency cancer outcomes such as colorectal cancer. These discrepancies highlight the complex and context-dependent effects of Asa on colorectal carcinogenesis, which may be influenced by age, duration of exposure, comorbidities, genetic predisposition, and tumor molecular profile. Importantly, the absence of favorable modulation of NF-κB, Ki67, or pS6K observed with Asa in ASAMET does not contradict the robust randomized evidence supporting Asa’s role in colorectal cancer prevention and prognosis. Rather, these findings suggest that Asa’s anticancer effects may be mediated predominantly through biological mechanisms not captured by the epithelial tissue biomarkers assessed in this study. In particular, increasing evidence indicates that Asa exerts clinically relevant anticancer activity through platelet-dependent pathways, including inhibition of thromboinflammation, modulation of platelet–tumor cell interactions, and enhancement of antitumor immune surveillance. Notably, recent mechanistic work has demonstrated that Asa can prevent metastatic dissemination by relieving platelet-derived TXA_2_–mediated suppression of cytotoxic T-cell function, thereby promoting immune-mediated clearance of disseminating tumor cells (14). Within this context, the lack of a joint effect of Asa and Met on mucosal proliferation does not preclude complementary or additive effects on colorectal cancer prevention or prognosis, as the two agents may act on distinct, nonoverlapping carcinogenic pathways.

The increase in Ki67 expression observed in right-sided colonic mucosa among patients receiving the AsaMet combination was unexpected and warrants further investigation. Although the biological relevance of this finding is unclear, it may reflect site-specific epithelial responses, compensatory hyperproliferation, or pharmacologic interactions between the two agents. It is not known whether the excess of colorectal cancer in the ASPREE trial was global or in a specific colon site, so inference with our findings is not possible. The absence of changes in global or segmental pS6K expression suggests limited modulation of mTORC1 signaling in normal mucosa at the doses used. This is noteworthy given Met’s known effects on AMPK–mTOR signaling in preclinical models and may indicate that pS6K is a less sensitive biomarker in the context of non-neoplastic tissue. Our results are consistent with those of Zell and colleagues (26) on the lack of modulation of pS6Ser235/236 or Ki67 immunostaining levels after 12 weeks of Met treatment among obese individuals with a history of colorectal adenoma. This suggests that Met in colorectal cancer mucosa of humans has no direct growth-inhibitory action via AMPK activation that leads to inhibition of mTOR activation and protein synthesis and reduced proliferation.

Toxicity was manageable and in line with the known profile of both drugs. Importantly, we did not observe any increase of gastrointestinal events observed in previous studies of patients treated with Met after colorectal cancer surgery, in whom tolerance to Met limited compliance (approximately 50% of the participants took at least 75% of the planned dosages; ref. 27).

Several limitations must be acknowledged. The trial was underpowered because of disruption in accrual (COVID-19) and early closure because of financial constraints, limiting the precision of estimates and the ability to detect modest biomarker changes. The relatively short follow-up precludes conclusions about the impact of these biomarker modulations on clinical outcomes such as recurrence or survival. As with most tissue-based biomarker studies, sampling errors can affect the results; for example, proliferation can differ within different regions of the large intestine and a single snapshot in time cannot account for larger temporal effects. Additionally, tissue quality varied dramatically between various sites which affected the downstream analyses. Finally, all participating centers were European, which may limit the generalizability of our findings to other populations (Supplementary Table S4).

Despite these limitations, this exploratory study has important strengths. To our knowledge, it is the first placebo-controlled trial to investigate the combined effects of Met and Asa on colorectal mucosal biomarkers in survivors of cancer. The use of paired pre- and posttreatment tissue samples, rigorous IHC quantification, and adjustment for colon side provide robust evidence of drug effects on the normal colonic microenvironment.

In conclusion, Met significantly reduced NF-κB expression in normal colonic mucosa of survivors of colorectal cancer, supporting its further evaluation as a potential chemopreventive agent. Asa did not demonstrate favorable biomarker modulation in this setting and may even promote mucosal proliferation under specific conditions. Results do not preclude effects of Asa on carcinogenesis through other complementary biological pathways. These findings emphasize the need for biomarker-driven and molecularly stratified prevention strategies and support the inclusion of inflammation-related endpoints in future chemoprevention trials.

## Supplementary Material

Supplementary Table 1 Supplementary Table 1 shows baseline immunohistochemical expression of NFκB, EGFR, Ki67 and pS6K in normal right and left colonic mucosa by treatment arm.

Supplementary Table 2 Supplementary Table 2 shows the pooled-arm analyses isolating the effects of aspirin and metformin on global NFκB expression, adjusted for baseline values.

Supplementary Table 3 Supplementary Table 3 shows the pooled-arm analyses isolating the effects of aspirin and metformin on global Ki67 expression, adjusted for baseline values.

Supplementary Table 4 Supplementary Table 4 describes the representativeness of the study participants with respect to sex, age, race/ethnicity and geography.

## Data Availability

The data generated in this study are not publicly available because of patient privacy and consent restrictions but are available from the corresponding author upon reasonable request.

## Code Availability

The statistical code used to perform the analyses in this study is available from the corresponding author upon reasonable request.
