# Supplementary material for: Biomarker Modulation by Aspirin and Metformin for Colorectal Cancer Prevention: Results from the ASAMET Randomized Phase II Trial
Source: Cancer Res Commun. 2026 Nov 4;6(11):2593–601. doi: 10.1158/2767-9764.CRC-26-0556 (PMC13732397; doi:10.1158/2767-9764.CRC-26-0556)
Supplement: Supplementary file 1 [file crc-26-0556_supplementary_table_1_suppst1.docx]

Supplementary Table 1. IHC baseline expression levels of biomarkers in normal colonic tissue

|  | **AsaMet**  **(N = 14)** | **Asa**  **(N = 12)** | **Met**  **(N = 12)** | **Pla**  **(N = 13)** | ***p*-value** |
| --- | --- | --- | --- | --- | --- |
| **NFκB**  Right  Left | 54.5 [47.0 – 59.9]  44.7 [24.4 – 54.8] | 49.5 [38.4 – 55.9]  43.9 [34.2 – 47.3] | 57.0 [48.6 – 59.2]  49.9 [47.8 – 52.3] | 51.6 [44.2 – 63.0]  38.3 [23.9 – 43.2] | 0.64  0.18 |
| **EGFR**  Right  Left | 0.09 [0.04 – 0.27]  0.40 [0.04 – 1.02] | 0.06 [0.01 – 0.11]  0.08 [0.02 – 0.16] | 0.08 [0.02 – 0.16]  0.13 [0.03 – 0.15] | 0.11 [0.08 – 0.15]  0.32 [0.06 – 0.55] | 0.61  0.15 |
| **Ki67**  Right  Left | 21.8 [16.3 – 35.1]  22.1 [17.3 – 32.4] | 27.7 [24.9 – 29.7]  20.2 [17.5 – 24.2] | 26.2 [22.5 – 28.7]  18.5 [11.7 – 25.3] | 24.2 [21.8 – 28.7]  15.3 [12.6 – 20.5] | 0.79  0.22 |
| **pS6K**  Right  Left | 74.2 [56.0 – 88.0]  77.1 [64.5 – 86.4] | 74.2 [64.3 – 81.3]  83.4 [62.1 – 84.5] | 77.0 [59.3 – 85.5]  79.4 [73.9 – 84.2] | 71.0 [59.9 – 83.4]  76.4 [75.0 – 78.7] | 0.99  0.84 |
| Median (Interquartile Range); the biomarkers are expressed as % positive cells. | | | | | |
