# Supplementary material for: Biomarker Modulation by Aspirin and Metformin for Colorectal Cancer Prevention: Results from the ASAMET Randomized Phase II Trial
Source: Cancer Res Commun. 2026 Nov 4;6(11):2593–601. doi: 10.1158/2767-9764.CRC-26-0556 (PMC13732397; doi:10.1158/2767-9764.CRC-26-0556)
Supplement: Supplementary file 2 [file crc-26-0556_supplementary_table_2_suppst2.docx]

Supplementary Table 2. Effect of ASA and MET regimens on global NFκB expression levels

| **NFkB global** | **Coef.** | **Std. Err.** | **t** | **P>\|t\|** | **95% Conf. Interval** |
| --- | --- | --- | --- | --- | --- |
| Met + Pla  Asa + AsaMet | -1.14 | 3.30 | -0.34 | 0.732 | -7.74 to 5.47 |
| Asa + Pla  Met + AsaMet | -7.29 | 3.25 | -2.24 | 0.029 | -13.79 to -0.79 |
| Met + Pla vs Asa + AsaMet (to isolate ASA effect); Asa + Pla vs Met + AsaMet (to isolate MET effect); All models adjusted for baseline marker values. | | | | | |
