# Supplementary material for: Biomarker Modulation by Aspirin and Metformin for Colorectal Cancer Prevention: Results from the ASAMET Randomized Phase II Trial
Source: Cancer Res Commun. 2026 Nov 4;6(11):2593–601. doi: 10.1158/2767-9764.CRC-26-0556 (PMC13732397; doi:10.1158/2767-9764.CRC-26-0556)
Supplement: Supplementary file 3 [file crc-26-0556_supplementary_table_3_suppst3.docx]

Supplementary Table 3. Effect of ASA and MET regimens on global Ki-67 expression levels

| **Ki67 global** | **Coef.** | **Std. Err.** | **t** | **P>\|t\|** | **95% Conf. Interval** |
| --- | --- | --- | --- | --- | --- |
| Met + Pla  Asa + AsaMet | 7.08 | 3.58 | 1.98 | 0.053 | -0.10 to 14.25 |
| Asa + Pla  Met + AsaMet | -3.56 | 3.73 | -0.96 | 0.344 | -11.03 to 3.92 |
| Met + Pla vs Asa + AsaMet (to isolate ASA effect); Asa + Pla vs Met + AsaMet (to isolate MET effect); All models adjusted for baseline marker values. | | | | | |
