# Supplementary material for: Biomarker Modulation by Aspirin and Metformin for Colorectal Cancer Prevention: Results from the ASAMET Randomized Phase II Trial
Source: Cancer Res Commun. 2026 Nov 4;6(11):2593–601. doi: 10.1158/2767-9764.CRC-26-0556 (PMC13732397; doi:10.1158/2767-9764.CRC-26-0556)
Supplement: Supplementary file 4 [file crc-26-0556_supplementary_table_4_suppst4.docx]

Supplementary Table 4. Representativeness of study participants

| **Cancer type / stage** | Stage I–III colorectal cancer (CRC), post-surgical resection. |
| --- | --- |
| **Sex** | CRC affects both sexes with a slight male predominance (~55% globally). ASAMET enrolled 32 males (63%) and 19 females (37%), consistent with the known sex distribution of stage I–III CRC survivors. No sex-based exclusion criteria were applied. |
| **Age** | Median age at CRC diagnosis in Europe is approximately 67 years. ASAMET enrolled patients aged 18–80 years (mean ~67 years across treatment arms), reflecting the typical age distribution of CRC survivors in the adjuvant and post-adjuvant setting. |
| **Race/Ethnicity** | The trial was conducted exclusively at European centres (Genoa, Italy; Ljubljana, Slovenia; Vienna, Austria). The population is predominantly of White/European ancestry. Racial and ethnic diversity data were not systematically collected, consistent with European clinical trial practice at the time of enrolment (2017–2024). Underrepresentation of non-White and non-European patients is acknowledged as a limitation. |
| **Geography** | All three participating sites are European. Results may not be directly generalisable to non-European or non-Western populations, in whom CRC incidence, tumour biology, and treatment context may differ. |
| **Other considerations** | Patients with Lynch syndrome, inflammatory bowel disease (IBD), ECOG performance status >1, and diabetic patients on pharmacological treatment were excluded. The enrolled population represents a relatively fit, non-diabetic CRC survivor cohort eligible for adjuvant chemoprevention. |
| **Overall representativeness** | The study population is broadly representative of the European CRC survivor population eligible for post-adjuvant chemoprevention trials. The main limitation in representativeness is the exclusive European enrolment and the absence of systematic race/ethnicity data collection, which restricts generalisability to non-European populations. |
